# ATP Recycling Fuels Sustainable Glycerol 3-Phosphate Formation in Synthetic Cells Fed by Dynamic Dialysis

**DOI:** 10.1021/acssynbio.2c00075

**Published:** 2022-04-04

**Authors:** Eleonora Bailoni, Bert Poolman

**Affiliations:** Department of Biochemistry, Groningen Biomolecular Sciences and Biotechnology Institute & Zernike Institute for Advanced Materials, University of Groningen, Nijenborgh 4, 9747 AG Groningen, The Netherlands

**Keywords:** sustainable minimal metabolism, selectively open system, continuous-flow dialysis, ATP recycling, phospholipid headgroup synthesis, glycerol 3-phosphate

## Abstract

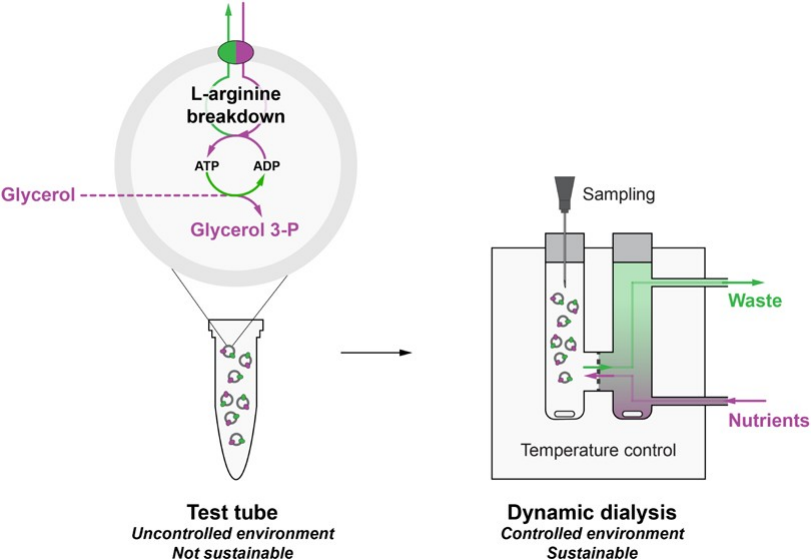

The bottom-up construction of an autonomously growing, self-reproducing cell represents a great challenge for synthetic biology. Synthetic cellular systems are envisioned as out-of-equilibrium enzymatic networks encompassed by a selectively open phospholipid bilayer allowing for protein-mediated communication; internal metabolite recycling is another key aspect of a sustainable metabolism. Importantly, gaining tight control over the external medium is essential to avoid thermodynamic equilibrium due to nutrient depletion or waste buildup in a closed compartment (*e.g.*, a test tube). Implementing a sustainable strategy for phospholipid biosynthesis is key to expanding the cellular boundaries. However, phospholipid biosynthesis is currently limited by substrate availability, *e.g.*, of glycerol 3-phosphate, the essential core of phospholipid headgroups. Here, we reconstitute an enzymatic network for sustainable glycerol 3-phosphate synthesis inside large unilamellar vesicles. We exploit the *Escherichia coli* glycerol kinase GlpK to synthesize glycerol 3-phosphate from externally supplied glycerol. We fuel phospholipid headgroup formation by sustainable l-arginine breakdown. In addition, we design and characterize a dynamic dialysis setup optimized for synthetic cells, which is used to control the external medium composition and to achieve sustainable glycerol 3-phosphate synthesis.

## Introduction

Synthetic biologists are joining forces internationally^1^ to build cellular mimics from the bottom up that stay away from thermodynamic equilibrium^2−5^ and display life-like properties, including physicochemical homeostasis,^6−8^ subcompartmentalization,^9−11^ sensing/communication,^12−14^ protein synthesis,^14,15^ DNA replication,^16,17^ energy and cofactor (re)generation,^11,15,18,19^ membrane growth,^20−23^ division,^24,25^ and Darwinian evolution.^26^ Synthetic cells are envisioned as selectively open systems where phospholipid bilayers maintain electrochemical gradients and provide spatial confinement to a sustainable minimal metabolism, while embedded membrane proteins promote directional communication and transport with the external environment.^2,3,6,25,27^ Importantly, internal recycling networks complementary to the protein-mediated exchange of nutrients and waste products are key to avoiding thermodynamic equilibrium.

Membrane expansion requires the generation of new membrane building blocks, *e.g.*, phospholipids and membrane proteins. In bacterial and eukaryotic cells, phospholipids consist of two acyl chains linked in positions 1 and 2 to a glycerol 3-phosphate moiety that is further decorated with functionalizing groups (for comprehensive reviews^28−30^). Thus, glycerol 3-phosphate represents an essential building block for membrane growth in (synthetic) cells. However, sustained phospholipid biosynthesis is currently limited by the availability of glycerol 3-phosphate, among other nutrients that do not diffuse through the phospholipid bilayer. To overcome depletion, an ATP-dependent glycerol kinase^31−34^ can be exploited to generate glycerol 3-phosphate from its membrane-permeable precursor, glycerol.

Metabolic energy conservation is essential for various processes in living and synthetic cells, including phospholipid biosynthesis. The l-arginine breakdown pathway is an enzymatic network for ATP recycling that has previously been used in a synthetic system to demonstrate physicochemical homeostasis coupled to the import of a compatible solute.^8,35^ This pathway consists of three cytosolic enzymes (ArcABC) that phosphorylate ADP into ATP by converting l-arginine into l-ornithine, CO_2_, and NH_4_^+^. The buildup of waste products is prevented by an antiporter (ArcD) that couples the import of l-arginine to the excretion of l-ornithine, whereas CO_2_ and NH_4_^+^ leave the cells by passive diffusion (NH_4_^+^ is in rapid equilibrium with NH_3_, which is membrane-permeable).

Maintaining an out-of-equilibrium state over a period sufficient for cellular growth and division ultimately requires tight control over the external medium composition. In a closed, noncontrolled environment (*e.g.*, a test tube), the nutrient concentrations decrease and waste products accumulate over time, and eventually, the system reaches equilibrium. In addition, the reconstitution of progressively more complex enzymatic networks in synthetic cells requires that metabolite levels are finely regulated to prevent depletion/accumulation bottlenecks caused by possible kinetics mismatches. Finally, batch reactions may be limited by the physicochemical properties of substrates (*e.g.*, poor solubility or instability, leading to aggregation or degradation) that would benefit from continuous feeding over time.

Dynamic dialysis (or continuous-flow dialysis) represents a convenient technology to generate an open environment and expose synthetic cells to tunable continuous feeding flows. As in batch dialysis, a filter of defined cutoff retains molecules above a certain size threshold, while smaller molecules are released into the exchanging solution, a continuously flowing solution in the case of dynamic dialysis.^36−45^ Continuous-flow dialysis offers unique advantages compared to other feeding techniques, namely: (i) the retention compartment can be accessed for sampling and performing instant perturbations; (ii) the sample of interest is not altered by tethering or other chemical modifications; and (iii) the retentate can be recovered for off-line analysis (*e.g.*, by liquid chromatography-mass spectrometry (LC-MS), high-performance liquid chromatography (HPLC), enzymatic assays, *etc.*). Nevertheless, dynamic dialysis devices have to date only been developed for *ad hoc* applications and thus suffer from limitations including (i) large sampling void volumes; (ii) detrimental gravity effects (*e.g.*, air bubble formation, pressure on dialysis filter, poor mixing, *etc.*); and (iii) physical stress on the retentate. To our knowledge, a customizable dynamic dialysis layout suitable for vesicle-based work has not been developed.

Here, we exploit the generation of metabolic energy by the arginine breakdown pathway to fuel sustainable phospholipid headgroup formation in synthetic cells. Further, we present a user-friendly modular dynamic dialysis setup optimized to continuously feed the cells with fresh medium. We characterize the dynamic dialysis setup and use it to achieve sustainable glycerol 3-phosphate synthesis.

## Results and Discussion

### Reconstitution of ATP Recycling and Glycerol 3-Phosphate Formation in Large Unilamellar Vesicles (LUVs)

**Figure 1:**
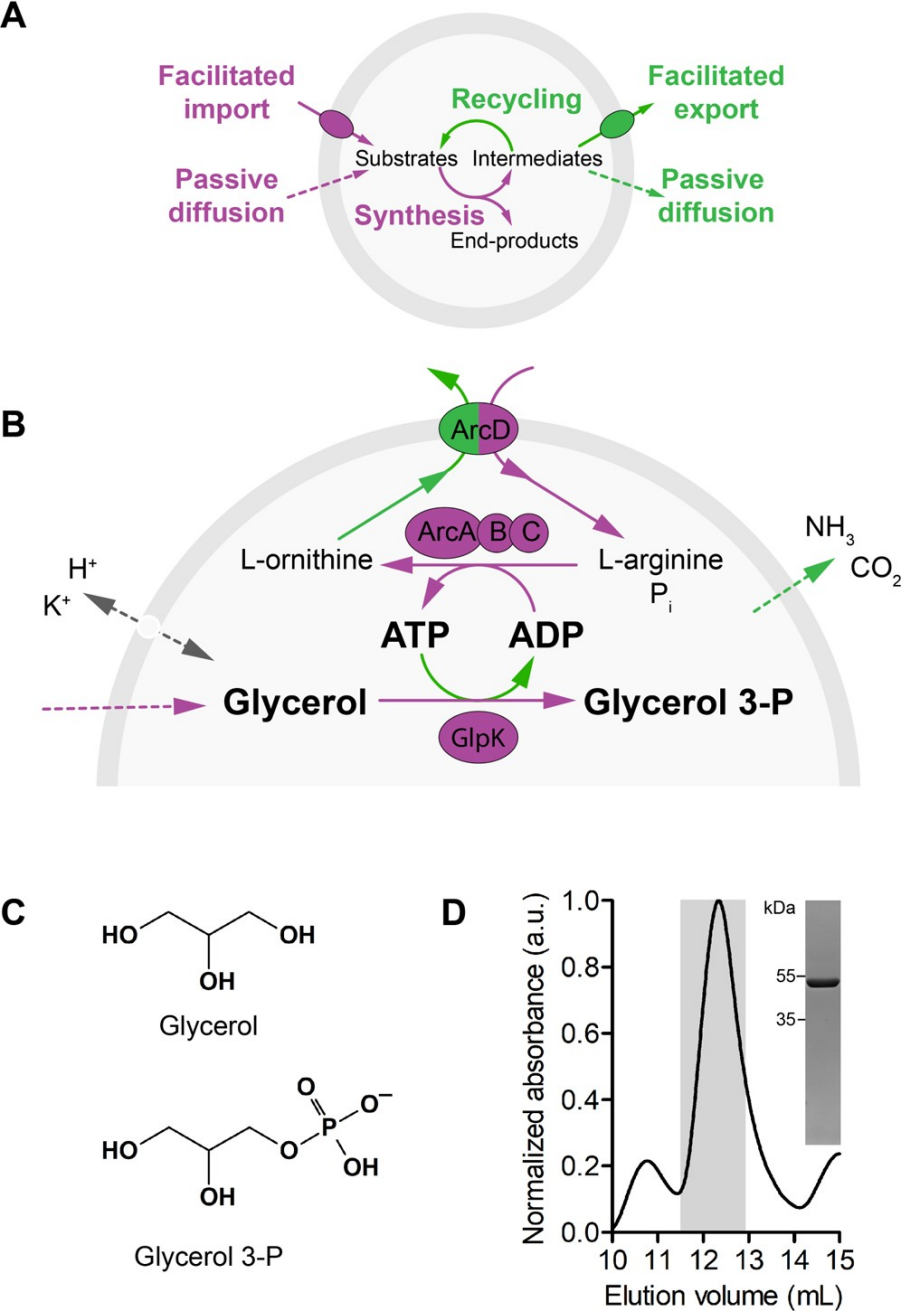
Sustainable glycerol 3-phosphate synthesis fueled by enzymatic ATP recycling. (A) Schematic of sustainable enzymatic network with recycling of metabolic energy and product formation. We envision a selectively open system where macromolecules are retained, while reactants are exchanged with the external medium either by membrane proteins (impermeable solutes) or by passive diffusion (permeable solutes) to maintain the system out of equilibrium for a prolonged period of time. Substrates are fed from the external medium (purple), while end-products are either recycled internally (cofactors) or exported (waste products) (green). The system ultimately reaches equilibrium if the external medium is not refreshed or infinitely large in volume, as is the case with vesicles in a test tube. (B) Schematic of enzymatic network for sustainable glycerol 3-phosphate synthesis, fueled by ATP generation and recycling via l-arginine breakdown. The arginine breakdown pathway is composed of an l-arginine/l-ornithine antiporter (ArcD2) and three cytosolic proteins (ArcA, ArcB, and ArcC1). In this system, l-arginine is imported from the external medium and used to phosphorylate ADP. The end-product l-ornithine is exported in exchange for l-arginine. Other end-products (CO_2_ and NH_3_) leave the vesicles passively according to their concentration gradient across the membrane bilayer. The vesicles carry the ionophores valinomycin and nigericin to dissipate any gradient of H^+^ and K^+^ or the possible formation of a membrane potential. (C) Chemical structure of glycerol and glycerol 3-phosphate. (D) Size-exclusion chromatogram and SDS-PAA gel image (inset) of purified *E. coli* GlpK (57 kDa).

We aimed to reconstitute out-of-equilibrium phospholipid headgroup formation in LUVs, an essential step toward implementing sustainable membrane growth within synthetic cells. To this end, we used the *Escherichia coli* glycerol kinase GlpK, an enzyme that converts glycerol into glycerol 3-phosphate at the expense of one molecule of ATP. Because glycerol permeates phospholipid membranes at high rates (permeability coefficient of 2.3 × 10^–5^ cm/s^46^), a dedicated glycerol importer is not required for glycerol 3-phosphate synthesis (Figure 1a,c).

In brief, we coupled glycerol kinase activity to a pathway for ATP generation based on the deamination of l-arginine (Figure 1b). For this, we overexpressed, purified, and reconstituted GlpK together with the components of the l-arginine breakdown pathway in LUVs composed of DOPE, DOPC, and DOPG at molar ratios of 1:2:1 (Figures 1a,b,d, S1, and S2). We used a lipid to membrane protein ratio of 400 (w/w) for the reconstitution of the l-arginine/l-ornithine antiporter ArcD2. We also included PercevalHR,^8,47^ an online ratiometric ATP/ADP sensor, in the lumen of the vesicles. We used 10 mM ADP, 0.5 mM l-ornithine to kickstart the antiporter, and 50 mM potassium phosphate (KPi), pH 7.0 as buffer inside the vesicles. The internal osmolality was 227 mOsm/kg, which was matched by 58 mM NaCl in the external medium that was further composed of 50 mM KPi pH 7.0 (Figure S3). In addition, we extensively washed the vesicles (three times in 6 mL of 50 mM KPi pH 7.0 plus 58 mM NaCl) to remove glycerol carried over from the enzyme stocks (see the Methods section). We used a combination of 1 μM nigericin plus 1 μM valinomycin (in DMSO, final 0.4% v/v) to dissipate any proton and potassium gradients and avoid artifacts in the ATP/ADP readout, owing to the pH sensitivity of PercevalHR.^47^

### ATP and ADP Quantification by Chemiluminescence

We assessed the performance of the arginine breakdown pathway by quantifying the ATP and the total nucleotide (ATP plus ADP) levels in the vesicles. To this end, we adapted the conventional luciferase assay developed for mammalian cells that was otherwise incompatible with our experimental setup due to the broad pH activity range and high stability of GlpK.^32^ Thus, we replaced the commercial alkaline stopping reaction with perchloric acid (PCA, final 1.16% v/v) plus ethylenediaminetetraacetic acid (EDTA, final 0.75 mM), which inactivated all enzyme activity. We then quenched PCA by mixing with an excess of KOH and KHCO_3_ (final concentration of 125 mM), which conveniently lead to perchlorate precipitation upon storage at −20 °C. Using an excess of KOH/KHCO_3_ resulted in a final pH of approximately 8, which was neutralized to approximately 7 by the addition of H_2_SO_4_ (final concentration of 7.7 mM) (Figure S4). While the commercial luciferase is active at alkaline pH, neutralization is important for the determination of the total nucleotide concentration, that is, in part of the sample where we converted ADP into ATP by the addition of pyruvate kinase (PK) and an excess of phosphoenolpyruvate (PEP, final concentration of 3.13 mM). We then used a commercial luciferase substrate solution to quantify ATP (plus ADP converted into ATP) by chemiluminescence.

We found that the total nucleotide levels amount to about 3 nmol for 0.1 mg total lipids, which corresponds to approximately 11 mM internal concentration, given a specific internal volume of 2.7 μL/mg total lipids^8^ (Figure S5). This value is in good agreement with the ADP concentration used in the reconstitution (10 mM), given the uncertainty in the internal volume measurements (*e.g.*, variation in vesicle size, multilamellarity).

**Figure 2:**
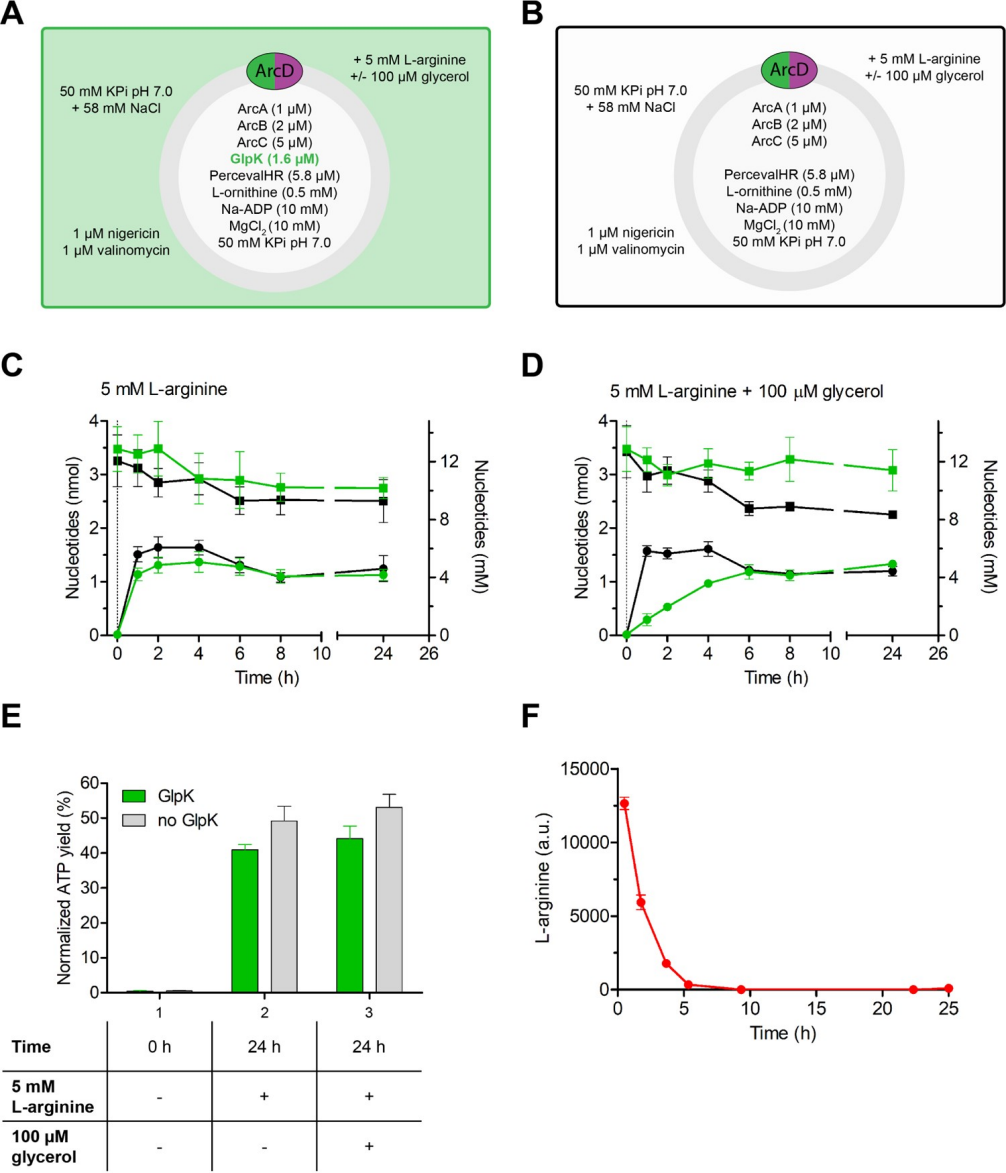
ATP and ADP quantification by chemiluminescence. (A) Schematic of the vesicle system including GlpK. (B) Schematic of the vesicles without GlpK. (C) ATP (dots) and total nucleotide levels (squares) in the absence of glycerol (*n* = 3; error bars represent s.e.m.). ATP synthesis was started by the addition of 5 mM l-arginine at *t* = 0. The conversion plateaus within 1 h, both in the presence (green) and absence (black) of GlpK. (D) ATP (dots) and total nucleotide levels (squares) in the presence of 100 μM glycerol (*n* = 3; error bars represent s.e.m.). ATP and glycerol 3-phosphate synthesis is started by the addition of 5 mM l-arginine plus 100 μM glycerol at *t* = 0. In the presence of GlpK (green), ATP buildup is slowed down by the concomitant synthesis of glycerol 3-phosphate. The kinetics are unchanged in the absence of GlpK (black). (E) Normalized ATP yield (*n* = 3; error bars represent s.e.m.). Each datapoint was normalized to the corresponding total nucleotide level (set as 100%). Only 50% of total ADP has been converted into ATP after 24 h, suggesting that the fraction of active vesicles is 50%. (F) l-Arginine depletion over time. HPLC data showing depletion of 5 mM l-arginine from the external medium. The rapid depletion is due to internal conversion (l-arginine breakdown to ornithine, CO_2_ plus NH_4_^+^) and the previously reported^24^ external conversion of l-arginine into l-citrulline plus NH_4_^+^due ArcA sticking to the external surface of the vesicles.

When 5 mM l-arginine was added to the system, ADP was converted into ATP, a process reaching a plateau within 1 h, which was followed by a drop in ATP that correlated with a decrease in the total nucleotide pool (Figure 2a–c). Importantly, no significant difference was observed when GlpK was left out from the system, indicating that the residual glycerol levels from the enzyme stocks were negligible. Next, we added 100 μM glycerol, which corresponds to 13.7–27.4 mM glycerol 3-phosphate if all glycerol would be converted inside the vesicles and assuming a total internal volume of 0.73–0.37% v/v, respectively, for the population of active vesicles (Figure S6). Because only 10 mM ADP was initially present in the vesicles, at least one full cycle of ADP conversion and recycling must have occurred. In the presence of glycerol, complete ATP recovery was observed after 6 h (Figure 2d). As expected, the kinetics of ATP formation was not affected by glycerol in the absence of GlpK.

The estimates of the internal volume probably have an error of about 10% (using the calcein fluorescence/quenching method^48^), but the uncertainty in the fraction of active vesicles is larger. It has been observed in numerous reconstitution and encapsulation studies that not all vesicles get the same amount of enzyme and even a significant fraction of giant unilamellar vesicles is typically devoid of any enzymatic activity despite the very large volume.^49−51^ In our reconstitution we incorporated one membrane protein and encapsulated four different enzymes and one fluorescence-based sensor, making it very likely that a fraction of the vesicles did not have all of the components and were not active. Moreover, the concentrations of protein that we encapsulated were in the range of 1–5 μM, which translated to 28–144 molecules per vesicle.^8^ Hence, stochastic effects will influence the activity and fraction of active vesicles. The arginine breakdown pathway produced 1.5 nmol ATP, corresponding to a normalized yield of active vesicles of approximately 50% (Figure 2e), which is remarkably high given the complexity of the vesicles we constructed.

### Online ATP/ADP Readout with PercevalHR

**Figure 3:**
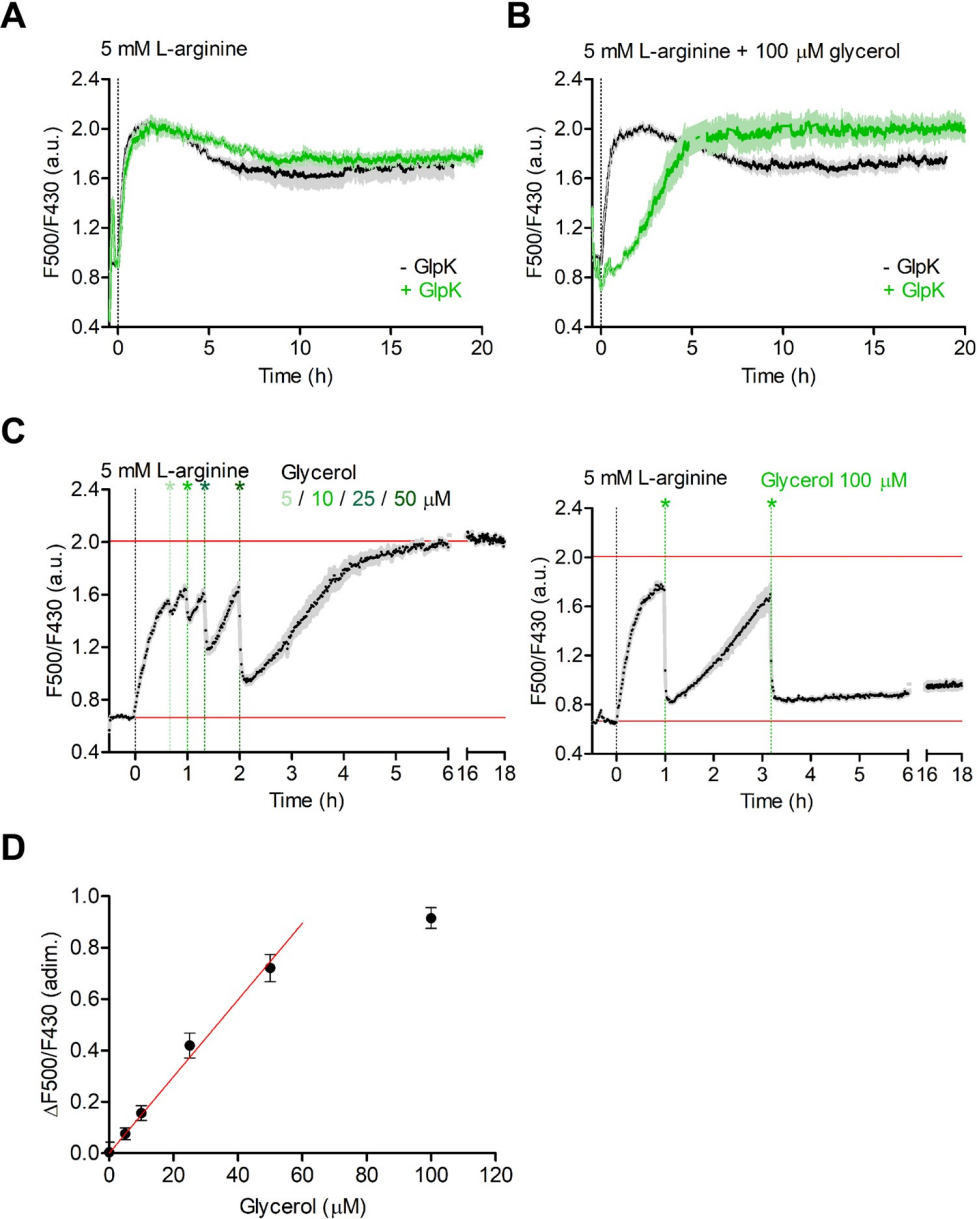
Online ATP/ADP readout with PercevalHR. (A) Apparent ATP/ADP ratio with (green) and without (black) GlpK in the absence of glycerol (*n* = 3; error bars represent s.e.m.). (B) Apparent ATP/ADP ratio with (green) and without (black) GlpK in the presence of 100 μM glycerol (*n* = 3; error bars represent s.e.m.). (C) Glycerol titration (*n* = 3; error bars represent s.e.m.). ATP synthesis is started by addition of 5 mM l-arginine at *t* = 0. Next, glycerol is added (i) in 0–50 μM steps (left) and (ii) in two 100 μM steps (right). (D) Change in the F500/F430 ratio before and after glycerol addition (*n* = 3; error bars represent s.e.m.). The data linearly correlates with the glycerol levels up to 50 μM glycerol (slope = 0.01492 ± 0.0004686).

We qualitatively exploited the online ATP/ADP readout by PercevalHR (excitation spectra, apparent F500/F430) as an on-line tool for the characterization of the pathway and for the monitoring of glycerol 3-phosphate synthesis. Upon addition of 5 mM l-arginine, the relative ATP/ADP levels increased with kinetics comparable to what was observed with the chemiluminescence assay (Figures 3a and S7). Similarly to the drop in ATP in the chemiluminescence assay, we observed that the ATP/ADP ratio decreased over time and stabilized at a value of approximately 90% after ∼8 h. The amino acid analysis by HLPC revealed that l-arginine was completely depleted at the time the ATP/ADP ratio stabilized (Figure 2f); here, we noted that a large part of the l-arginine was converted externally into l-citrulline due to residual ArcA bound to the vesicle surface.^8^l-Citrulline is also transported by ArcD2 in exchange for internal l-ornithine, albeit at a much lower rate.^35^ Addition of a 3-fold higher concentration of l-arginine (15 mM) enhanced the ATP/ADP decrease over time (Figure S8). The control without GlpK ruled out a possible effect of this enzyme. A control with a 10-fold excess of nigericin plus valinomycin (10 μM) excluded the possibility that proton and or potassium gradients were formed and affected the readout of PercevalHR fluorescence (Figure S9). Also, the external pH was stable under our experimental conditions (Figure S10). We do not currently have a complete explanation for the small but significant decrease in the ATP levels detected by chemiluminescence and in the ATP/ADP ratio measured with PercevalHR. However, it is possible for the vesicles to leak small molecules such as ATP over time.

The kinetics of ATP/ADP buildup are in good agreement with the chemiluminescence data also in the presence of 100 μM glycerol (14 mM internal volume) (Figures 3b and S6). Strikingly, the presence of glycerol stabilized the plateau ATP/ADP signal. Next, we quantified glycerol 3-phosphate formation by titrating increasing amounts of glycerol after full ADP conversion: (i) 0–50 μM glycerol steps, estimated total internal concentration of 24.6 mM for the “active” volume (Figure 3c, left), and (ii) two additions of 100 μM glycerol, estimated total internal concentration of 54.8 mM for the “active” volume (Figure 3c, right). Under the assumption of instantaneous conversion of glycerol into glycerol 3-phosphate and measuring the changes in F500/F430, we observed a linear dependence of the PercevalHR readout and the amount of glycerol added up to 50 μM (Figure 3d). Upon addition of 100 μM glycerol, ATP was fully hydrolyzed due to rapid consumption by GlpK and recycling by the arginine breakdown pathway became limiting. The recovery of ATP/ADP ratio was ultimately hindered by the decrease in internal phosphate concentration and l-arginine depletion; we have 50 mM inorganic phosphate inside the vesicles and thus active vesicles can maximally form 50 mM glycerol 3-phosphate.

Together, the chemiluminescence and fluorescence data highlight the limitation of closed compartments for the development of sustainable metabolic networks, namely that the external medium composition is not constant and in the here presented metabolic network inorganic phosphate becomes depleted. The core constitution of a phosphate transporter would settle the bottleneck of depletion of inorganic phosphate. In the present setup, a large excess of glycerol cannot be used, as the rapid glycerol 3-phosphate formation depletes the ATP pool, thereby disturbing the metabolic energy homeostasis. Rather, a constant feed with a low concentration of glycerol would sustain the glycerol 3-phosphate synthesis longer. Similarly, a constant feed of (an excess of) l-arginine would prevent depletion by the external ArcA activity, and thus keep the system away from equilibrium for a longer period of time.

### Design of a Modular Dynamic Dialysis Setup for Metabolic Networks in Vesicles

To provide the synthetic cells with a constant environment in terms of substrate (glycerol and l-arginine) and product (l-citrulline, l-ornithine, NH_4_^+^, and CO_2_) levels, we developed a modular continuous-flow setup that overcomes the limitations of previously reported geometries^36−45^ and has been optimized for working with LUVs.

**Figure 4:**
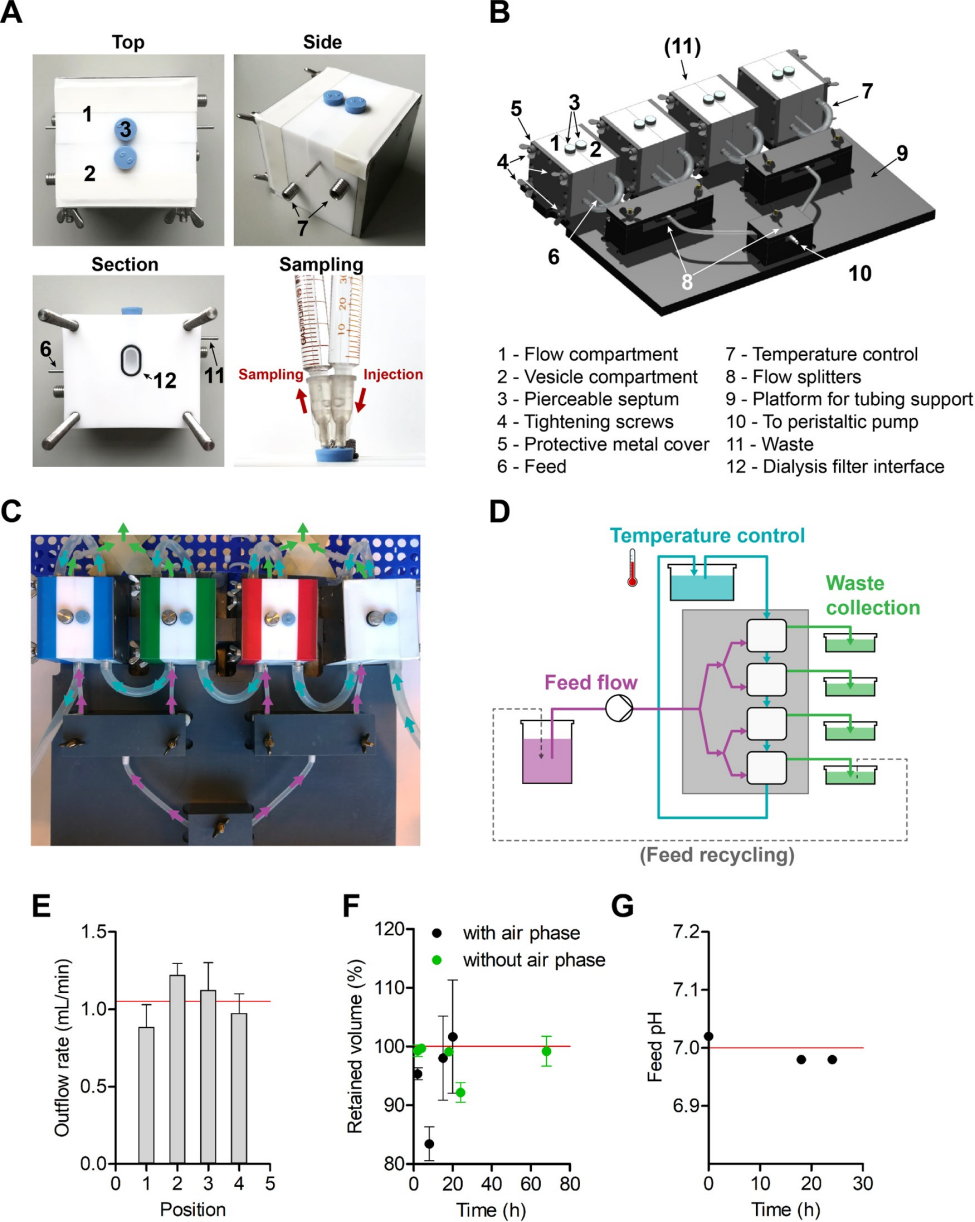
Multichamber continuous-flow setup optimized for synthetic cells. (A) Continuous-flow chamber as a modular unit of the multichamber setup. The chamber is composed of two compartments, one dedicated to the vesicles and the other to the feed flow. The compartments are sealed at the top with pierceable rubber stoppers. Sampling occurs by means of two syringes, to avoid volume changes. (B, C) Overview of a four-chamber continuous-flow setup in a parallel arrangement. All components are carefully secured to a supporting platform to ensure a constant feed flow. (D) Schematic representation of the flows. A peristaltic pump feeds the nutrient solution to the dialysis chambers (purple). The waste (green) is collected in dedicated beakers and may optionally be recycled into the feeding solution. An independent water flow (light blue) through dedicated jackets allows us to regulate the temperature. (E) Averaged outflow rate of the feed solution at each position (*n* = 4; error bars represent s.e.m.). (F) Volume in the vesicle compartment over time (*n* = 2; error bars represent s.e.m.). Sealing septa (black) promote the formation of an air phase and introduce significant volume changes. Instead, pierceable stoppers (green) fill the compartment opening and reduce the volume variability. (G) Feed pH. The pH of a feed composed of 5 mM l-arginine in 50 mM KPi pH 7.0 is constant over time.

The central unit of our continuous-flow system is a chamber composed of two symmetrical halves carved out of a block of poly(tetrafluoroethylene) (PTFE), a material that is chemically inert in aqueous solutions and a good insulator. The PTFE blocks are laterally supported by metal plaques to minimize deformation. The two halves host cylindrical compartments (volume of 1.2 mL), interfaced with one another through a lateral window hosting a vertically oriented dialysis filter (Figures 4a and S11). One of the two compartments is dedicated to the vesicle sample, while the second one is intended for the feeding solution. The second compartment is further equipped with lateral openings, *i.e.*, with an inlet at the base and an outlet at the top, to ensure optimal flow circulation. Importantly, these openings are parallel to the dialysis window so that the vesicles are subjected to a minimal physical stress coming from the medium flow. Stirring bars are added for gentle mixing to enhance metabolite diffusion.

### Flow Rates

For higher throughput, we connected four continuous-flow chambers in parallel (Figure 4b–d). The feeding solution was flowed to the chambers by a peristaltic pump through a spliced tubing system. We found that even a minor misalignment in the tubing system significantly increased the variability in the flow rates of each chamber. Hence, we carefully secured the tubing to a supporting platform with dedicated cases.

The flow rate is set by the peristaltic pump and is easily changed over the course of an experiment with a minor lag time (second range). The (change in) flow rate is ultimately affected by the overall backpressure of the system. While a single chamber could be fed at a slow flow rate (100–200 μL/min), four chambers in a parallel configuration required a higher flow rate (0.5–2.5 mL/min). We assessed the outflow rate of our working setup by permutating the chambers relative to each other and measuring the outflow volume over time. We found that the outflow rates are comparable for all devices at each position, with an average outflow rate of 1.1 ± 0.2 mL/min (Figures 4e and S12). Importantly, because a large excess of volume was used for the feeding solution (100 times the volume of the vesicle compartment and about 10 000 times the internal vesicle volume), we opted to recycle medium when expensive substrates or prolonged timescales were required.

### Volumes and Sampling

Next, we checked that the volume in the chamber compartments remains constant under the applied flow rate. We found that sealing septa allow the formation of a compressible air phase that caused an undesired ∼20% volume change over 20 h (Figure 4f). When we replaced the septa with pierceable stoppers and carefully filled the compartments to the top, we found that the variation was reduced to ∼5% over 68 h, a time frame much longer than the duration of an experiment.

To prevent the formation of negative pressure inside the vesicle compartment, sampling was performed by simultaneously replacing the sample volume with a fresh solution. This approach introduces a dilution of the sample components that does not affect ratiometric measurements (*e.g.*, with PercevalHR) but affects other methods. We thus asked whether absolute quantification is possible. To this end, we filled the vesicle compartment with 500 μM NADH in 50 mM KPi pH 7.0 and collected samples by volume replacement with buffer. We found the theoretical and measured dilution factors to be in good agreement (Figure S13).

### Temperature Control

The flow dialysis chambers are equipped with jackets dedicated to temperature control by an independent water flow connected to a thermostatic water bath. When the flow dialysis chambers are connected serially with respect to the water flow, we measured a small drop in temperature between the first and the last device, which is prevented by parallel tubes from the water bath. In addition, we found that PTFE well insulates the compartments, as approximately 30 ± 5 min (*n* = 2) were required to lower the temperature by 5 °C upon an instantaneous change in the temperature of the water bath. Finally, we confirmed that bacterial growth does not occur in the flow device, as indicated by the clear appearance of the feeding solution and a constant pH of 7.0 ± 0.2 over 24 h (Figure 4g).

### Vesicle Retention and Metabolite Equilibration

Choosing a suitable dialysis filter is of paramount importance for the continuous-flow setup. The dialysis filter should be chemically inert, retain the vesicles and allow metabolite diffusion at good rates. To this end, we used track-etched polycarbonate membranes with a pore diameter of 50 nm. These filters are highly compatible with phospholipid vesicles and have a monodisperse pore size distribution;^52^ in addition, they are suitable for our LUVs with an average vesicle radius of approximately 85 nm.^8^

**Figure 5:**
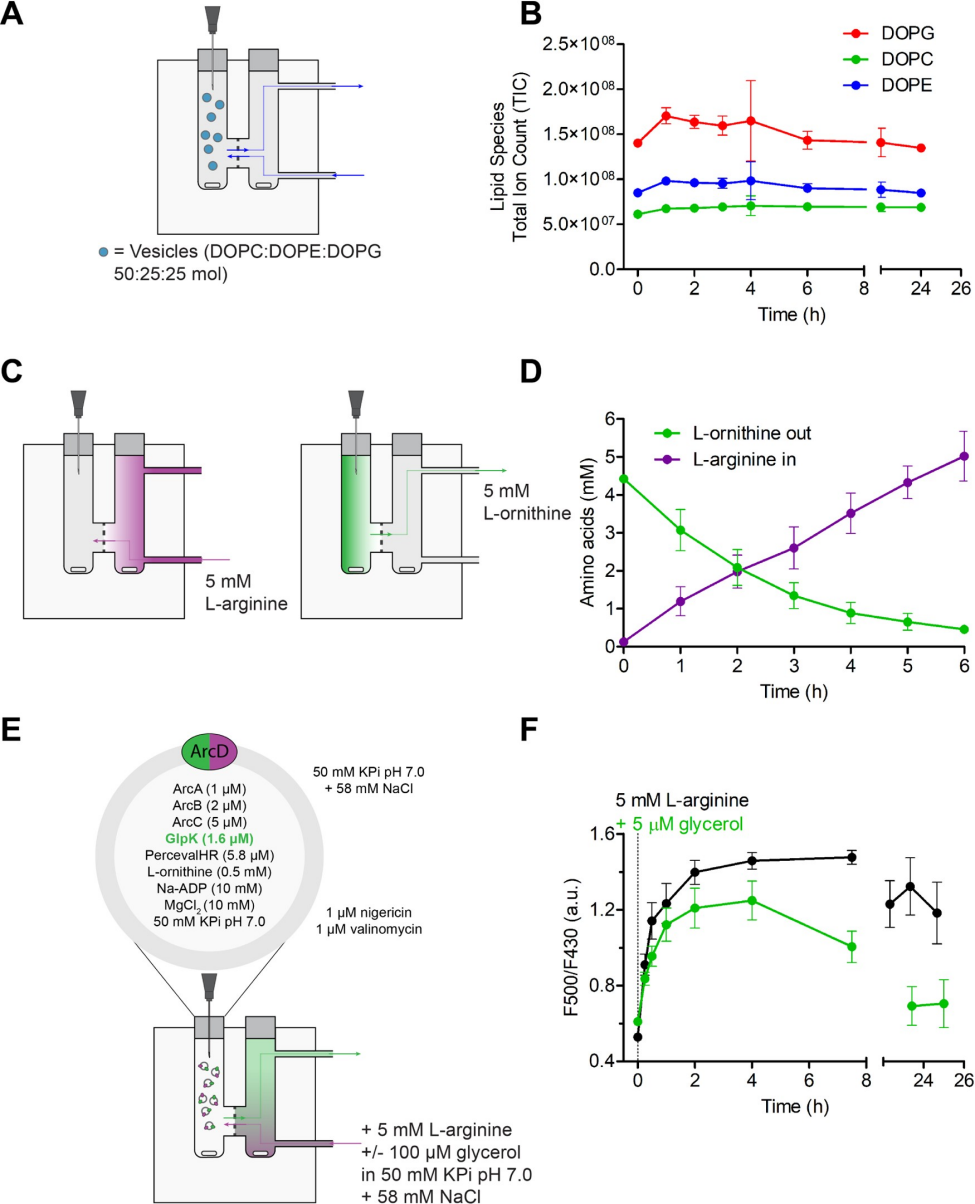
ATP and glycerol 3-phosphate formation with continuous substrate feed. (A, B) Vesicles are retained by the polycarbonate filter (50 nm pore diameter). (A) Schematic of the experimental setup; 2.78 mg/mL of vesicles are added to the vesicle compartment and sampled over time. A flow of 50 mM KPi pH 7.0 is applied to the feed compartment. (B) LC-MS data normalized for dilution reveals that the total lipid composition is constant over time (*n* = 2; error bars represent s.e.m.). (C, D) Metabolites equilibrate through the polycarbonate filter. (C) Schematic of the experimental setup. A metabolite gradient is imposed by applying 5 mM l-arginine/l-ornithine/l-citrulline in 50 mM KPi pH 7.0 to either of the chamber compartments. (D) HPLC data normalized for dilution reveal metabolite equilibration through the polycarbonate filter (*n* = 4; error bars represent s.e.m.). Equilibration occurs at the same rate for both compartments. (E, F) ATP and glycerol 3-phosphate synthesis with a continuous l-arginine and glycerol feed. (E) Schematic of the experimental setup. The vesicles are applied to the vesicle compartment and the substrates are fed through the feed compartment. (F) Online ATP/ADP readout measured with PercevalHR (*n* = 3; error bars represent s.e.m.).

We checked that the vesicles are retained by applying 2.78 mg/mL empty vesicles (25:50:25 mol DOPG/DOPC/DOPE) in the presence of the polycarbonate filter and a flow of 50 mM KPi pH 7.0. We collected vesicle samples (60 μL) over time and quantified the total lipid content by LC-MS. We found that the total lipid content normalized for dilution was constant over 24 h (Figure 5a,b), indicating that the vesicles were effectively retained by the polycarbonate filter.

Next, we demonstrated nutrient feeding and waste product removal across the polycarbonate filter. To this end, we imposed a metabolite concentration gradient by applying 5 mM l-arginine, 5 mM l-ornithine, and 5 mM l-citrulline (in 50 mM KPi pH 7.0) either to the vesicle compartment or to the feeding solution. The HPLC analysis of the samples collected from the vesicle compartment revealed that all amino acids diffused across the polycarbonate filter along their concentration gradient and reached equilibrium after 6 h (Figures 5c,d and S14).

### ATP and Glycerol 3-Phosphate Formation with a Continuous l-Arginine and Glycerol Feed

We applied the vesicles with reconstituted glycerol kinase and arginine breakdown pathway to our continuous-flow device. Under a continuous 5 mM l-arginine feed, we observed that the ATP/ADP ratio plateaus after 2 h (Figure 5e,f) and slightly decreases after 24 h, a behavior consistent with what we have seen in the batch experiment. When we introduced a 5 μM glycerol feed, ATP synthesis became significantly slower due to the concomitant glycerol 3-phosphate synthesis. Strikingly, the ATP/ADP level dropped to the initial value after 24 h, indicating that the vesicles were depleted of ATP. We attributed this to the fact that continuous glycerol 3-phosphate synthesis in our setup is ultimately limited by the phosphate availability provided by the internal buffer.

Overall, we have demonstrated that our continuous-flow setup is successful in providing the synthetic vesicles with a constant medium where (diluted) substrates are constantly fed and waste products are removed. This allows us to tune the metabolic networks and thus maintain the synthetic cells away from equilibrium over a longer period of time. To our knowledge, this is the first continuous-flow setup described that is suitable for working with LUVs and studies of metabolic networks.

## Conclusions

Sustainable synthesis of glycerol 3-phosphate inside synthetic vesicles is an important milestone toward full phospholipid biosynthesis. We have designed an enzymatic pathway for continuous glycerol 3-phosphate formation inside lipid vesicles with l-arginine and glycerol administration as variables. We used absolute and relative ATP quantification techniques to indirectly estimate glycerol 3-phosphate synthesis, and we found the system to be limited only by substrate depletion. To provide the synthetic cells with a constant medium composition, we designed and optimized a continuous-flow dialysis setup, allowing for continuous l-arginine feed and waste product drain. We believe that the vesicle-optimized dynamical system could find wider application in synthetic biology by maintaining other compartmentalized enzymatic networks out-of-equilibrium over long timescales.

## Methods

### Chemicals and Media

Ampicillin sodium salt (Carl Roth); ATPlite chemiluminescence Assay System (PerkinElmer); deoxyribonuclease I from bovine pancreas (Sigma); dimethyl sulfoxide (DMSO) (Sigma); dipotassium hydrogen phosphate trihydrate (Merck); ethylenediaminetetraacetic acid (EDTA) dipotassium salt dihydrate (Sigma); glycerol (Boom); imidazole (Carl Roth); isopropyl-β-d-thiogalactopyranoside (IPTG) (Thermo Scientific); l-(+)-arabinose (Sigma); l-arginine (Sigma); l-citrulline (Sigma); l-ornithine monohydrochloride (Carl Roth); Lysogeny Broth Miller (Formedium); magnesium chloride hexahydrate (Sigma); magnesium sulfate heptahydrate (Merck); Neodisher Alka 800 (Dr. Weigert); Ni-NTA Agarose (Qiagen); perchloric acid 60% (Sigma); phenylmethyl sulphonyl fluoride (PMSF) (Carl Roth); phosphoenolpyruvate (PEP) trisodium salt heptahydrate (Carl Roth); potassium bicarbonate (Sigma); potassium dihydrogen phosphate (Merck); potassium hydroxide, ca. 85%, for analysis, pellets (Acros Organics); pyruvate kinase/lactic dehydrogenase from rabbit muscle (Sigma); sodium chloride (Merck); Terrific Broth (Formedium); sulfuric acid (H_2_SO_4_) (Boom); 1,2-dioleoyl-*sn*-glycero-3-phospho-(1′-*rac*-glycerol) (DOPG) (sodium salt, powder) (Avanti Polar Lipids); 1,2-dioleoyl-*sn*-glycero-3-phosphocholine (DOPC) (powder) (Avanti Polar Lipids); 1,2-dioleoyl-*sn*-glycero-3-phosphoethanolamine (DOPE) (powder) (Avanti Polar Lipids); adenosine 5′-triphosphate disodium salt trihydrate (Roche); adenosine 5′-diphosphate sodium salt (Sigma); valinomycin (Sigma); and Nigericin sodium salt (Sigma).

### Plasmids

#### ArcA, ArcB, ArcC1, ArcD2, and PercevalHR

The plasmids used for ArcA, ArcB, ArcC1, and cysteine-less ArcD2 (C395T-C487T double mutant) overexpression were as previously reported.^8^ PercevalHR was cloned from the original vector (Addgene plasmid #49081^47^) into pBAD24 (Table S1) for expression from the l-arabinose promoter, with the sequence specifying a 7×-His tag added to the 5′ end of the gene and sequenced (Eurofins Genomics; Figure S1).

#### GlpK

The *glpK* sequence was PCR-amplified from the genome of *E. coli* BL21-DE3 with the PfuX7 polymerase (produced in-house) and overlapping primers containing a uracil base (Eurofins Genomics, Table S1). The PCR-amplification product was ligated in a pRSETA backbone according to the uracil excision protocol.^53^ The final vector contains an IPTG-inducible T7 promoter, a 6×-His tag at the N-terminus followed by the *glpK* coding sequence, and a cassette for ampicillin resistance. The vector sequence was checked by DNA sequencing (Eurofins Genomics; Figure S1).

### Protein Overexpression and Cell Lysis

#### ArcX

ArcA, ArcB, ArcC1, and ArcD2 were overexpressed in *Lactococcus lactis* as previously reported^8^ with the following differences: (i) cells were washed with 100 mM KPi pH 7.5 (instead of 100 mM KPi pH 7.0); (ii) cells were resuspended in 50 mM KPi pH 7.5 plus 10% v/v glycerol (instead of 50 mM KPi pH 7.0); and (iii) a 10 L culture was used for ArcD2 overexpression (instead of 2 L). The cells were lysed as reported except that 1 mM PMSF was added before and not after disruption. Membrane vesicles were prepared as previously reported for ArcD2.

#### PercevalHR

A colony from *E. coli* MC1061 cells transformed with pBADPercevalHR was used to start a 150 mL preculture with lysogeny broth and 100 μg/mL ampicillin (LB-amp) and incubated o/n at 37 °C, 200 rpm. Next, the preculture was used to start four 500 mL cultures in baffled flasks: two in terrific broth (TB-amp) and two in LB-amp. These were grown at 37 °C, 200 rpm until an OD_600_ of 0.5 was reached, after the temperature was lowered to 18 °C. PercevalHR expression was then induced after 45 min with 0.01% l-arabinose. After 48 h of induction at 18 °C, the cells were harvested by centrifugation (15 min, 6000*g*, 4 °C). The cells were washed with 50 mM KPi pH 7.5, weighed, and resuspended together in 50 mM KPi pH 7.5 with 10% v/v glycerol (20 g cells in total, 250 g/L). Cells were flash-frozen and stored at −80 °C for later use. Before lysis, the cells were thawed in a water bath at room temperature and diluted to 200 g/L; 100 μg/mL DNase, 1 mM PMSF, and 2 mM MgCl_2_ were also added. Lysis was performed with a single passage through an HPL6 press (Maximator GmbH) at 20 KPsi at 4 °C. Next, 5 mM EDTA was added, and the lysate was cleared by ultracentrifugation (45 min, 150 000*g*, 4 °C). The supernatant was flash-frozen and stored at −80 °C for later use.

#### GlpK

The vector pRSETA-GlpK was freshly transformed into chemically competent *E. coli* BL21-DE3, plated onto LB-amp plates, and incubated o/n at 37 °C. A colony was used to start an LB-amp preculture that was grown o/n at 37 °C, 200 rpm. A large-scale culture was prepared by 1:100 dilution of the preculture into 1 L fresh LB-amp medium and incubated at 37 °C, 200 rpm. Induction was performed with 0.1 mM IPTG at an OD_600_ of 0.6; incubation was prolonged o/n at 18 °C. Cells were harvested by centrifugation (15 min, 6000*g*, 4 °C), washed with 100 mM KPi pH 7.0, and centrifuged again. The cell pellet was resuspended in 50 mM KPi pH 7.0 to a final OD_600_ of 140, flash-frozen with liquid nitrogen, and stored at −80 °C. For lysis, the cells were gently thawed in a water bath at room temperature, after which 2 mM MgSO_4_, 100 μg/mL DNase plus 1 mM PMSF were added. The cells were filtered to remove residual cotton cloth and passed two times through an HPL6 press (Maximator GmbH) at 20 KPsi at 4 °C. The disrupted cells were centrifuged for 60 min at 145 000*g* at 4 °C, then the supernatant was collected, flash-frozen with liquid nitrogen, and stored at −80°C.

### Protein Purification

#### Soluble Proteins

All soluble proteins (ArcA, ArcB, ArcC, PercevalHR, and GlpK) were purified following a standardized protocol. A volume of 10 mL of cell lysate (100 < OD_600_ < 200) was quickly thawed in a water bath at room temperature and then transferred to ice. A volume of 2 mL (1 CV) of Ni-NTA agarose was washed with 24 CVs Milli-Q water and 4 CVs of equilibration buffer (50 mM KPi pH 7.5, 200 mM KCl, 10% v/v glycerol, 10 mM imidazole). Next, 10 mM imidazole was added to the cell lysate and this was incubated with the preequilibrated Ni-NTA agarose beads at 4 °C with nutation. After 1 h, the flow through was removed and the beads were washed with 20 CVs of washing buffer (50 mM KPi pH 7.5, 200 mM KCl, 10% v/v glycerol, 50 mM imidazole). The proteins were eluted in consecutive steps (60% CV first step, followed by 40% CV steps) with elution buffer (50 mM KPi pH 7.5, 200 mM KCl, 10% v/v glycerol, 500 mM imidazole). 5 mM K-EDTA was added to the elution fractions. The elution samples were then loaded onto a Superdex 200 Increase 10/300 GL column (Ge Healthcare) preequilibrated with storage buffer (50 mM KPi pH 7.0, 100 mM KPi, 10% v/v glycerol). The peak fractions were pooled together and concentrated with an Amicon Ultra-2 Centrifugal Filter Unit (Millipore) of proper cutoff (30 or 50 kDa) to a final concentration of 5–10 mg/mL. The concentrated proteins were aliquoted in 20 μL samples to minimize freeze-thawing, flash-frozen in liquid nitrogen, and stored at −80 °C.

#### ArcD2 Purification and Reconstitution

The procedure for ArcD2 purification and reconstitution into pre-formed liposomes was as previously reported,^8^ albeit with a different lipid composition (25:50:25 mol DOPE/DOPC/DOPG).

### Encapsulation of the Arginine Breakdown Pathway with GlpK

To a 1.5 mL empty vial were added in the following order: 10 mM Na-ADP (dissolved in 50 mM KPi pH 7.0); 50 mM KPi pH 7.0, 10 mM MgCl_2_ (dissolved in Milli-Q water); 0.5 mM l-ornithine (dissolved in 50 mM KPi pH 7.0); 37.5 μg ArcA (1 μM); 96 μg ArcB (2 μM); 72 μg ArcC1 (5 μM); 71 μg PercevalHR (5.8 μM) and 35.8 μg GlpK (1.6 μM). This suspension was carefully mixed and transferred onto one aliquot of pre-formed ArcD2 proteoliposomes (66.6 μL, 6.66 mg total lipids, 25:25:50 mol DOPG/DOPE/DOPC, lipid/ArcD2 400:1, 50 mM KPi pH 7.0), prepared as described.^8^ The two suspensions were gently resuspended and mildly vortexed to ensure proper mixing. Next, five freeze–thaw cycles were performed by flash-freezing with liquid nitrogen, and subsequently, the samples were transferred to a water bath at about 10 °C. The proteoliposomes were then either used immediately or stored in liquid nitrogen for later use (in this case, the last thawing step was performed at the moment of use). The proteoliposomes were extruded 13× through Nuclepore Track-Etched Membranes with 400 nm pore diameters (Whatman, GE Healthcare) preequilibrated with 0.5 mM l-ornithine, 10 mM Na-ADP and 10 mM MgCl_2_ in 50 mM KPi pH 7.0. The proteoliposomes were then diluted into 6 mL of 50 mM KPi pH 7.0 with 58 mM NaCl and washed by centrifugation (25 min, 325 000*g*, 4 °C). The washing procedure was performed three times (total 18 mL). Next, the proteoliposomes were resuspended to a volume of 1.2 mL (5.55 mg/mL) to which 1 μM valinomycin plus 1 μM nigericin was added from a concentrated DMSO stock (500 μM, final DMSO = 0.4% v/v). The proteoliposomes were used immediately or stored at 4 °C up to 72 h.

### Estimation of Residual Glycerol Concentration upon Vesicle Washing

We estimated the glycerol level present in the vesicle samples as a result of the encapsulation of enzymes stored in 10% v/v (1.37 M) glycerol. We reconstituted approximately 50 μL of proteins (sum of volumes of ArcA, ArcB, ArcC, GlpK, and PercevalHR) in 200 μL encapsulation volume, which resulted in a final 342.5 mM glycerol. After extrusion, the liposomes were washed three times with 6 mL of buffer, which diluted the glycerol to 3.8 mM (dilution factor of 90×). We assumed an average pellet volume of 40 μL (166.67 mg/mL total lipids) that, when diluted to the working concentration of 2.7 mg/mL total lipids, would result in maximally 60 μM glycerol (dilution factor 60×). In practice, we find hardly any glycerol carried over from the enzyme stock solutions.

### Internal ATP and Total Nucleotide (ATP Plus ADP) Quantification by Chemiluminescence

The proteoliposomes were diluted 1:1 to a final concentration of 2.7 mg/mL with 50 mM KPi pH 7.0 plus 58 mM NaCl and were incubated at 30 °C. Next, the reaction was started by addition of 5 mM l-arginine (from a 500 mM stock in 50 mM KPi pH 7.0) or 5 mM l-arginine plus 100 μM glycerol (from a 3.9 mM stock in 50 mM KPi pH 7.0). Samples of 100 μL were collected over a period of 25 h and immediately mixed with 20 μL of 7% v/v PCA and 4.5 mM EDTA (1% v/v PCA with 643 μM EDTA, final volume 140 μL). The samples were diluted with 20 μL of 50 mM KPi pH 7.0 + 58 mM NaCl (final volume 120 μL) and 20 μL of 1 M KOH + 1 M KHCO_3_ to quench PCA (125 mM, final volume 160 μL). Timepoints at 0 h (before l-arginine and glycerol addition) were also prepared. Calibration curves with ATP or ADP were prepared accordingly from 0–0.2 mM ATP or ADP stocks (in 50 mM KPi plus 58 mM NaCl) using 120 μL (0–24 nmol). To promote optimal KClO_4_ precipitation, samples were immediately incubated o/n at −20 °C. Next, the samples were thawed and the precipitated salt was pelleted with a tabletop centrifuge (10 min, RT). A volume of 60 μL was removed from the supernatant twice (for ATP and for total nucleotide quantification) from each sample, including the calibration curve (0–9 nmol ATP or ADP in technical replicate). To all samples were then added 7.7 mM H_2_SO_4_ (5 μL from a 100 mM stock, final volume 65 μL), 15.63 mM MgCl_2_ (5 μL from a 500 mM stock, final volume 160 μL) and 80 μL of 50 mM KPi pH 7.0 + 58 mM NaCl to a final volume of 160 μL. In addition, 3.13 mM PEP (5 μL from a 100 mM stock) and 2.4–4 U PK/LDH were added only to the total nucleotide samples (and ADP calibration curve), while 20 μL of Milli-Q water were added to the ATP samples instead. The samples were incubated for 3 h at 37 °C and then transferred to the wells of a 96-well, white, F-bottom microplate 655095 (Greiner Bio-One). To each well were then added 50 μL of substrate solution from the ATPlite chemiluminescence Assay System. The plate was shortly shaken, dark-adapted for 20 min and the chemiluminescence was read in an FL600 Microplate Fluorescence Reade (BioTek).

### l-arginine Levels by HPLC

The same samples used for the quantification of ATP by chemiluminescence were also used for detecting l-arginine levels by HPLC. Samples with a volume of 20 μL were quenched with PCA and neutralized as above; derivatization and HPLC analysis were performed as previously reported.^24^

### Online ATP/ADP Readout with PercevalHR

A volume of 120 μL of 2.7 mg/mL liposomes was pipetted into an Ultra-Micro-cuvette 105.252-QS (Hellma Analytics) and incubated at 30 °C for 30 min in an FP-8300 spectrofluorometer (Jasco). Fluorescence spectra of PercevalHR were collected over time (excitation = 400–520 nm, bandwidth = 5 nm; emission = 550 nm, bandwidth = 5 nm). The reaction was started by the addition of 5 mM l-arginine (dissolved in 50 mM KPi pH 7.0). Glycerol (dissolved in 50 mM KPi pH 7.0) was added at different timepoints in steps of 0–100 μM.

### Design and Assembly of a Multichamber-Flow Dialysis Setup

#### Setup of Four Chambers in a Parallel Configuration

The following parts were assembled together: (1) four in-house designed continuous-flow chambers; (2) an in-house designed supporting platform; (3) a Masterflex Peristaltic Tubing Pump (Cole-Parmer); (4) a water thermostat (Julabo). Norprene Food L/S Precision Pump Tubing 06402-14 (Masterflex) was used to connect the peristaltic pump to the supporting platform. Plastilab Tapered Y-Shaped Tubing Connectors 7052700 (Kartell) were used to sequentially split the flow into four subflows to flexible silicon tubing. The outlet silicon tubing system was connected with two Y-shaped tubing connectors to form two final streams. To minimize the flow rate variations, all Y-shaped tubing connectors were fixed to hosting cases onto the supporting board. The flow device was operated onto two adjacent stirring plates.

#### Cleaning and Sampling Procedure

The following protocol was used before and after an experiment. At this time, the water bath was not connected to the flow device. All chambers were assembled without a dialysis filter and washed twice with 500 mL of Neodisher (4 mL/L). When an experiment was performed, the Neodisher solution was flushed out and the device was washed twice with 500 mL of Milli-Q water filtered through PTFE filters with a pore diameter of 2 μm (Whatman, GE Healthcare); buffers used in the experimental procedure were all filtered accordingly. For sampling, 100 μL gastight syringes 1710 LT SYR (Hamilton) were used per continuous-flow chamber equipped with FINE-JECT injection needles, 27G × 3/4 – 0, 40 mm × 20 mm (Henke-Sass, Wolf). One syringe served for suction, the other for injection. In-between samples collection, the syringes were cleaned with Neodisher and mQ, while the needles were replaced.

### Technical Validation of Flow Device

#### Outflow Rate Determination

The outflow rate was determined by assembling the continuous-flow setup with polycarbonate membranes with pore diameters of 50 nm (Avestin) and applying a water flow. Water samples were collected every 2 min from the outlet tubing and weighted. This procedure was repeated by permutating all chambers.

#### Volume in the Vesicle Compartment

The volume present in the vesicle compartment was measured over time in the same setup as for the outflow rate determination. At each timepoint, the feed flow was stopped and the water present in each vesicle compartment was carefully removed and weighed. This experiment was performed with Precision Seal rubber septa Z553956 (Sigma) and with pierceable rubber stoppers (6 × 10, 18 mm, Saint-Gobain Performance plastics), after which the rubber stoppers were always used.

#### Feed pH

A feeding solution (5 mM l-arginine in 50 mM KPi plus 58 mM NaCl) was applied to the continuous-flow setup and the pH of the waste beaker was measured over time.

#### Liposome Retention

Liposome retention was checked by equipping the continuous-flow chambers with 50 nm polycarbonate filters (Avestin), preequilibrated with 50 mM KPi pH 7.0. Empty liposomes (1.2 mL, 2.7 mg/mL total lipids in 50 mM KPi pH 7.0) were added to the vesicle compartments. A 60 μL timepoint was taken at *t* = 0, then the liposomes were subjected to the flow and sampled up to 25 h. The samples were diluted with 100 μL of 50 mM KPi pH 7.0 and analyzed by LC-MS as previously reported.^54^

#### Metabolite Diffusion

The continuous-flow chambers with 50 nm polycarbonate filters (Avestin) were preequilibrated with 50 mM KPi pH 7.0. Next, an amino acid solution (5 mM l-arginine, 5 mM l-ornithine, 5 mM l-citrulline in 50 mM KPi pH 7.0) was added either to the feed flow (diffusion-in) or to the vesicle compartments (diffusion-out). In both cases, the other compartment contained 50 mM KPi pH 7.0 to generate a concentration gradient. Samples of 60 μL were collected, derivatized, and analyzed by HLPC as previously reported.^8^

### ATP and Glycerol 3-Phosphate Synthesis in the Continuous-Flow Setup

The continuous-flow chambers were equipped with 50 nm polycarbonate filters (Avestin) and a 30 °C water bath connected and gentle stirring was applied and preequilibrated with filtered 50 mM KPi pH 7.0 plus 58 mM NaCl. Next, the liposomes (1.2 mL, 5.55 mg/mL total lipids in 50 mM KPi pH 7.0 plus 58 mM NaCl) were added to the vesicle compartments. Timepoints at *t* = 0 were taken, after which the flow was replaced with 500 mL of feeding solution (filtered 50 mM KPi pH 7.0, 58 mM NaCl, 5 mM l-arginine ±5 μM glycerol). Timepoints were taken up to 25 h: external buffer was used for volume replacement in the first 6 h, while the feeding solution was used afterward. In addition, the feeding solution was replaced with 500 mL of fresh solution after 6 h to minimize the chance of bacterial growth in the continuous-flow device. The relative ATP/ADP ratio was measured with an FP-8300 spectrofluorometer (Jasco) with the following procedure: first, Ultra-Micro-cuvettes 105.252-QS (Hellma Analytics) were prewarmed at 30 °C with 70 μL of buffer (external buffer up to 6 h, then feeding solution); to these, 60 μL samples were added; and fluorescence spectra of PercevalHR were rapidly acquired (see above).

## Supporting Information Available

Sequences of glpK *and* PercevalHR; primers used to clone glpK into pRSETA and percevalHR into pBAD24; size-exclusion profiles and SDS-PAA gel of purified proteins; osmolality of internal solution and calibration for compensation of external medium; calibration curves for pH determination in the chemiluminescence assay; estimation of internal nucleotide concentration in the chemiluminescence assay; estimation of internal glycerol concentration; calibration curve of PercevalHR in solution and in liposomes; ATP/ADP in the presence of 15 mM l-arginine; ATP/ADP in the presence of a 10-fold excess of valinomycin and nigericin; pH of external medium over time; continuous-flow chamber; estimation of dilution factor introduced by sampling; outflow feed rate of continuous-flow setup; and equilibration of metabolites through a 50 nm polycarbonate filter (PDF)

## Abbreviations

ADP: adenosine diphosphate
ArcA: l-arginine deiminase
ArcB: l-ornithine transcarbamoylase
ArcC: carbamate kinase
ArcD: l-arginine/l-ornithine exchanger
ATP: adenosine triphosphate
DMSO: dimethyl sulfoxide
DOPC: dioleoyl phosphatidylcholine
DOPE: dioleoyl phosphatidylethanolamine
DOPG: dioleoyl phosphatidylglycerol
EDTA: ethylenediaminetetraacetic acid
GlpK: glycerol kinase
Glycerol: 3-P glycerol 3-phosphate
HPLC: high-performance liquid chromatography
LC-MS: liquid chromatography–mass spectrometry
LUV: large unilamellar vesicles
PCA: perChloric acid
PK/LDH: pyruvate kinase/lactate dehydrogenase
