# Supplementary material for: ATP Recycling Fuels Sustainable Glycerol 3-Phosphate Formation in Synthetic Cells Fed by Dynamic Dialysis
Source: ACS Synth Biol. 2022 Apr 4;11(7):2348–60. doi: 10.1021/acssynbio.2c00075 (PMC9295154; doi:10.1021/acssynbio.2c00075)
Supplement: Supplementary file 1 — sb2c00075_si_001.pdf [file sb2c00075_si_001.pdf]

Supporting Information

ATP recycling fuels sustainable glycerol 3-phosphate formation in synthetic cells fed by dynamic dialysis

Eleonora Bailoni[1] and Bert Poolman[1*]

[1]Department of Biochemistry, Groningen Biomolecular Sciences and Biotechnology Institute & Zernike Institute for Advanced Materials, University of Groningen, Nijenborgh 4, 9747 AG, Groningen, The Netherlands

*To whom correspondence should be addressed (email: b.poolman@rug.nl)

Keywords: sustainable minimal metabolism | selectively open system | continuous-flow dialysis | ATP recycling | phospholipid headgroup synthesis | glycerol 3-phosphate

Table of contents

Figure S1) Sequences of *glpK* and *percevalHR* gene

Figure S2) Size-exclusion profiles and SDS-PAA gel of purified proteins

Figure S3) Osmolality of internal solution and calibration for compensation of external medium

Figure S4) Calibration curves for pH determination in the chemiluminescence assay

Figure S5) Estimation of internal nucleotide concentration in the chemiluminescence assay

Figure S6) Estimation of internal glycerol concentration

Figure S7) Calibration curve of PercevalHR in solution and in vesicles

Figure S8) ATP/ADP in the presence of 15 mM L-arginine

Figure S9) ATP/ADP in the presence of a tenfold excess of valinomycin and nigericin

Figure S10) pH of external medium over time

Figure S11) Continuous-flow chamber

Figure S12) Outflow feed rate of continuous-flow setup

Figure S13) Estimation of dilution factor introduced by sampling

Figure S14) Equilibration of metabolites through a 50 nm polycarbonate filter

Table S1) Primers used to clone *glpK* into pRSETA and *percevalHR* into pBAD24

Figure S1 | Sequences of *glpK* and *percevalHR*. A) *glpK*. T7 polymerase promoter (grey); spacing (black); ribosome-binding site (blue); translation initiation codon (green); 6x-His tag (purple); *E. coli glpK* (orange); stop codon (red). B) *percevalHR*. pBAD (grey); spacing (black); ribosome-binding site (blue); translation initiation codon (green); 7x-His tag (purple); *percevalHR* (orange); stop codon (red).

A) glpK

5'TAATACGACTCACTATAGGGAGACCACAACGGTTTCCCTCTAGAAATAATTTTGTTTAACTTTAAGAAGGAGATATACATATGCATC
ATCATCACCATCATATGACTGAAAAAAAATATATCGTTGCGCTCGACCAGGGCACCACCAGCTCCCGCGCGGTCGTAATGGATCAC
GATGCCAATATCATTAGCGTGTCGCAGCGCGAATTTGAGCAAATCTACCCAAAACCAGGTTGGGTAGAACACGACCCAATGGAAAT
CTGGGCCACCCAAAGCTCCACGCTGGTAGAAGTGCTGGCGAAAGCCGATATCAGTTCCGATCAAATTGCAGCTATCGGTATTACGA
ACCAGCGTGAAACCACTATTGTCTGGGAAAAAGAAACCGGCAAGCCTATCTATAACGCCATTGTCTGGCAGTGCCGTCGTACCGCA
GAAATCTGCGAGCATTTAAAACGTGACGGTTTAGAAGATTATATCCGCAGCAATACCGGTCTGGTGATTGACCCGTACTTTTCTGGC
ACCAAAGTGAAGTGGATCCTCGACCATGTGGAAGGCTCTCGCGAGCGTGCACGTCGTGGTGAATTGCTGTTTGGTACGGTTGATAC
GTGGCTTATCTGGAAAATGACTCAGGGCCGTGTCCATGTGACCGATTACACCAACGCCTCTCGTACCATGTTGTTCAACATCCATAC
CCTGGACTGGGACGACAAAATGCTGGAAGTGCTGGATATTCCGCGCGAGATGCTGCCAGAAGTGCGTCGTTCTTCCGAAGTATAC
GGTCAGACTAACATTGGCGGCAAAGGCGGCACGCGTATTCCAATCTCCGGGATCGCCGGTGACCAGCAGGCCGCGCTGTTTGGT
CAGTTGTGCGTGAAAGAAGGGATGGCGAAGAACACCTATGGCACTGGCTGCTTTATGCTGATGAACACTGGCGAGAAAGCGGTGA
AATCAGAAAACGGCCTGCTGACCACCATCGCCTGCGGCCCGACTGGCGAAGTGAACTATGCGTTGGAAGGTGCGGTGTTTATGGC
AGGCGCATCCATTCAGTGGCTGCGCGATGAAATGAAGTTGATTAACGACGCCTACGATTCCGAATATTTCGCCACCAAAGTGCAAA
ACACCAATGGTGTGTATGTGGTTCCGGCATTTACCGGGCTGGGTGCGCCGTACTGGGACCCGTATGCGCGCGGGGCGATTTTCGG
TCTGACTCGTGGGGTGAACGCTAACCACATTATACGCGCGACGCTGGAGTCTATTGCTTATCAGACGCGTGACGTGCTGGAAGCG
ATGCAGGCCGACTCTGGTATCCGTCTGCACGCCCTGCGCGTGGATGGTGGCGCAGTAGCAAACAATTTCCTGATGCAGTTCCAGT
CCGATATTCTCGGCACCCGCGTTGAGCGCCCGGAAGTGCGCGAAGTCACCGCATTGGGTGCGGCCTATCTCGCAGGCCTGGCGG
TTGGCTTCTGGCAGAACCTCGACGAGCTGCAAGAGAAAGCGGTGATTGAGCGCGAGTTCCGTCCAGGCATCGAAACCACTGAGCG
TAATTACCGTTACGCAGGCTGGAAAAAAGCGGTTAAACGCGCGATGGCGTGGGAAGAACACGACGAATTAA3'

B) percevalHR

5'AAACCAATTGTCCATATTGCATCAGACATTGCCGTCACTGCGTCTTTTACTGGCTCTTCTCGCTAACCAAACCGGTAACCCCGCTT
ATTAAAAGCATTCTGTAACAAAGCGGGACCAAAGCCATGACAAAAACGCGTAACAAAAGTGTCTATAATCACGGCAGAAAAGTCCAC
ATTGATTATTTGCACGGCGTCACACTTTGCTATGCCATAGCATTTTTATCCATAAGATTAGCGGATCCTACCTGACGCTTTTTATCGC
AACTCTCTACTGTTTCTCCATACCCGTTTTTTGGGCTAACAGGAGGAATTAACCATGAAACATCACCATCACCATCACCATGGAGCTA
GCATGAAAAAGGTGGAATCCATCATCAGGCCCGAAAAGCTGGAGATCGTTAAGAAGGCTCTCTCGGACGCTGGATATGTGGGTATG
ACCGTCTCTGAAGTCAAAGGCTCTGGCGTCCAGGGCGGCATCTTCGAGAGGTACCGAGGAAGGGTGTACTCTGCAGGCTACAACA
GCGACAACGTCTACATCACCGCCGACAAGCAGAAGAACGGCATCAAGGCCAACTTCAAGATCCGCCACAACATCGAGGACGGCGG
CGTGCAGCTCGCCGACCACTACCAGCAGAACACCCCCATCGGCGACGGCCCCGTGCTGCTGCCCGACAACCACTACCTGAGCTT
CCAGTCCAAGCTGAGCAAAGACCCCAACGAGAAGCGCGATCACATGGTCCTGCTGGAGTTCGTGACCGCCGCCGGGATCACTCTC
GGCATGGACGAGCTGTACAAGGGCGGTTCCGGAGGCATGGTGAGCAAGGGCGAGGAGCTGTTCACCGGGGTGGTGCCCATCCT
GGTCGAGCTGGACGGCGACGTAAACGGCCACAAGTTCAGCGTGTCCGGCGAGGGCGAGGGCGATGCCACCTACGGCAAGCTGA
CCCTGAAGCTGATCTGCACCACCGGCAAGCTGCCCGTGCCCTGGCCCACCCTCGTGACCACCCTGGGCTACGGTCTCCAATGCTT
CGCCCGCTACCCCGACCACATGAAGCAGCACGACTTCTTCAAGTCCGCCATGCCCGAAGGCTACGTCCAGGAGCGCACCATCTTC
TTCAAGGACGACGGCAACTACAAGACCCGCGCCGAGGTGAAGTTCGAGGGCGACACCCTGGTGAACCGCATCGAGCTGAAGGGC
ATCGACTTCAAGGAGGACGGCAACATCCTGGGGCACAAGCTTGAGTACAACGGCACCATAGTAGATCTGATCCCTAAGGTAAAAAT
TGAACTAGTGGTGAAGGAGGAGGACGTCGATAACGTGATAGACATTATTTGCGAAAATGCCCGCACAGGTAACCCCGGCGACGGT
AAGATATTTGTGATCCCAGTGGAGCGAGTGGTCAGGGTGCGAACCAAAGAGGAGGGAGCATCTGGTGGTGGATCCGGTGGTGGC
GGTGCATCTGGTATGAAAAAGGTTGAAGCTATTATTCGCCCAGAGAAACTGGAGATTGTTAAGAAGGCCCTGTCCGACGCCGGCTA
TGTTGGCATGACCGTTTCCGAGGTTAAAGGCCGCGGTGCCGGCGGTGGCGACCTGATTCCTAAGGTGAAGATCGAGCTGGTTGTT
AAGGAGGAAGACGTTGATAATGTTATCGACATTATCTGCGAGAACGCCCGCACTGGCAATCCTGGTGACGGCAAAATCTTCGTTAT
ACCGGTCGAGCGAATTGTGCGAGTACGAACCAAAGAAGAGGGTGCCTCCGGCGGTGGCGGTGGCTCCGGTGGCGCCAGCGGCA
TGAAGAAGGTTGAGGCCATCATCCGCCCAGAGAAGCTGGAAATCGTTAAGAAGGCCTTAAACGACGACGGCTACGTCGGCATGAC
AGTTAGCGAGGTTAAAGGTCGCGGTGCCGGTGGGGGTGATCTGATTCCTAAGGTTAAAATCGAGCTCGTTGTTAAGGAGGAGGAC
GTTGACAACATTATCGATATCATCTGCGAAAACGCTCGCACCGGCAACCCAGGCGACGGCAAGATTTTCGTCATTCCTGTTGAGAG
AGTGGTGCGTCCCCGTACGAAGGAGGAGGGCAAGGAAGCACTGTGA3'

Table S1 | Primers used to clone *glpK* into pRSETA and *percevalHR* into pBAD24. The primers contain an uracil base required for uracil excision[1].

Gene	Name of primer	Sequence of primer (5' to 3')
glpK	glpK-fwd	ATCACCATCA**U**ATGACTGAAAAAAAATATATCGTTGCGCT
	glpK-rev	ATCAAGCT**U**TTATTCGTCGTGTTCTTCCCAC
	bck-fwd	AAGCTTGA**U**CCGGCTGCTAAC
	bck-rev	ATGATGGTGA**U**GATGATGCATATGTATATCTCC
percevalHR	prcHR-fwd	ACCATGAAACA**U**CACCATCACCATCAC
	prcHR-rev	AGATCACAG**U**GCTTCCTTGCCCTC
	bck-fwd	ACTGTGATC**U**CCAGCTTGGCTGTTTTG
	bckb-rev	ATGTTTCATGG**U**TAATTCCTCCTGTTAGCC

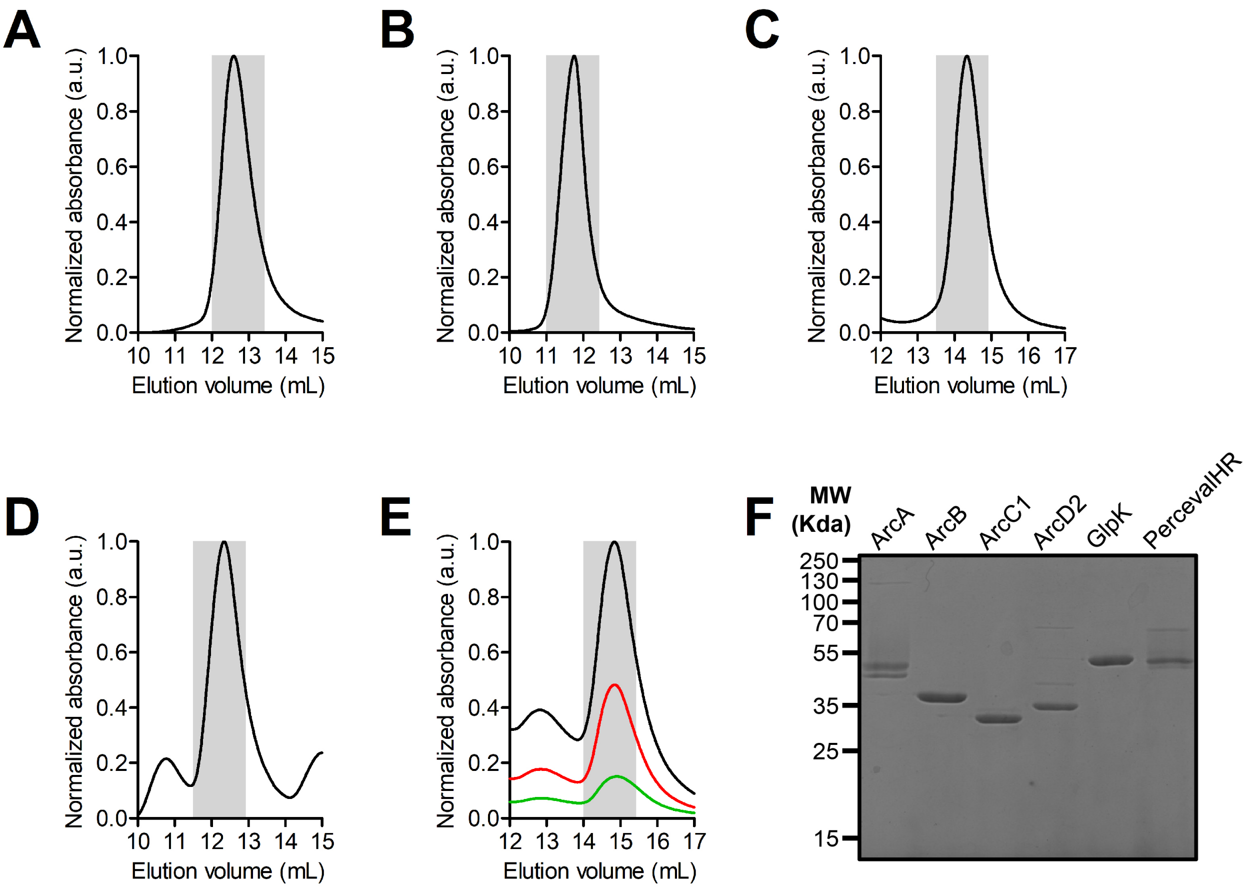

Figure S2 | Size-exclusion profiles and SDS-PAA gel of purified proteins. A-E) The normalized absorbance at 280 nm (black); 430 nm (red); 450 nm (red) is shown. Monomeric molecular weights are given in brackets. The peak areas highlighted are pooled together and used for experiments. A) ArcA (47 kDa[2,3]); B) ArcB (41 kDa[2,3]); C) ArcC1 (73 kDa[2,3]); D) GlpK (57 kDa[4]); E) PercevalHR (64 kDa[2,5]); F) SDS-PAGE gel of the proteins used in this study.

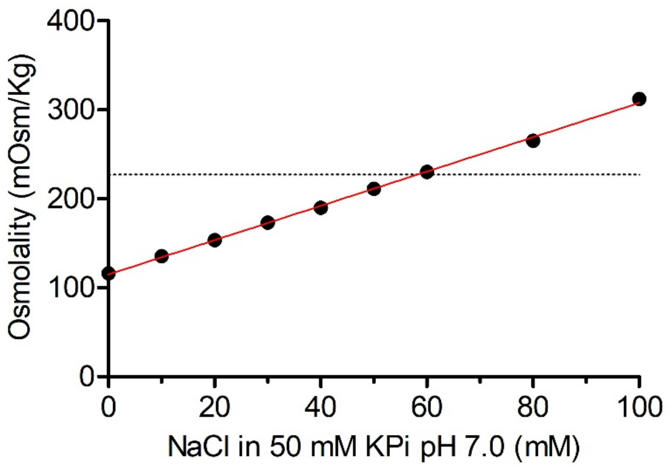

Figure S3 | Osmolality of internal solution and calibration for compensation of external medium. We measured the osmolality of the solution encapsulated in vesicles with a freezing point osmometer (Osmomat 3000 basic, Gonotec). We included the encapsulated components (1 µM ArcA, 2 µM ArcB, 5.8 µM PercevalHR, 10 mM Na-ADP, 10 mM $MgCl_2$, 0.5 mM L-ornithine) without pre-formed proteoliposomes (replaced with 50 mM KPi) and without ArcC, stored in 10% vol/vol glycerol (replaced with 50 mM KPi plus 100 mM KCl, under the assumption that glycerol is rapidly diluted out and that ArcC does not significantly contribute to osmolality). We measured an osmolality value of 227 mOsm/kg. We later stored all enzymes in 10% vol/vol glycerol, but since we did not assume a glycerol contribution to the osmolality, we did not repeat the measurement. We prepared a calibration curve of 50 mM KPi pH 7.0 with increasing concentrations of NaCl. From the linear fit (slope = 1.928 ± 0.02711; y(x0) = 114.9 ± 1.443), we found that 58 mM NaCl in 50 mM KPi pH 7.0 is required to match the internal osmolality.

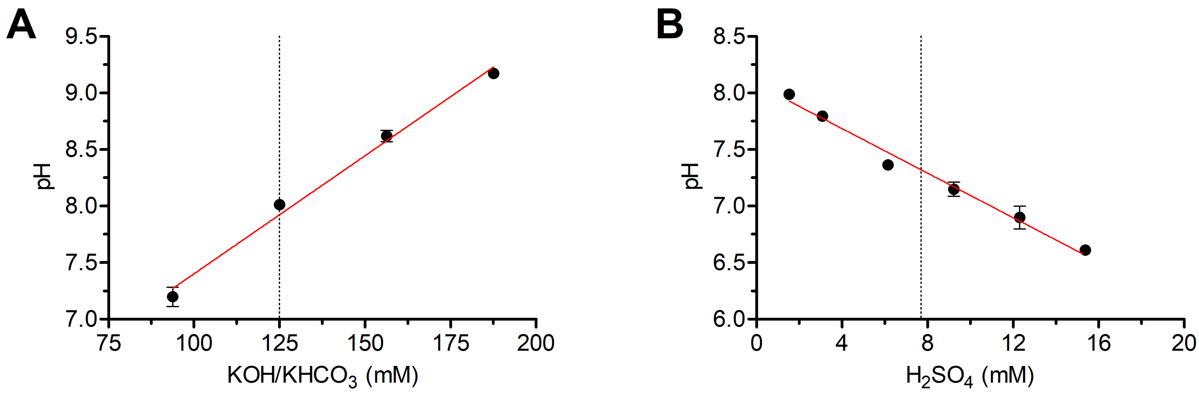

Figure S4 | Calibration curves for pH determination in the chemiluminescence assay. A) A solution of 1 M $KOH/KHCO_3$ was titrated into 60 uL 50 mM KPi pH 7.0 and then stored at -20 ˚C o/n. Upon complete $KClO_4$ precipitation, the salt was pelleted by a 10 minutes centrifugation step with a table top centrifuge at maximum speed. The supernatant was diluted with 200 uL milliQ water and the pH was measured with a pH electrode (ProLab 1000, SY Analytics). From the linear fit: slope = 0.02088 ± 0.001382; y(x0) = 5.314 ± 0.2002. The concentration of $KOH/KHCO_3$ used results in a pH of 8.0, thus ensuring complete PCA quenching. B) A solution of 100 mM H_2SO_4 is used to neutralize the pH of the supernatant, a step required for PK activity. From the linear fit: slope = -0.09840 ± 0.004955; y(x0) = 8.079 ± 0.04257. The concentration of H_2SO_4 used results in pH ~7.2.

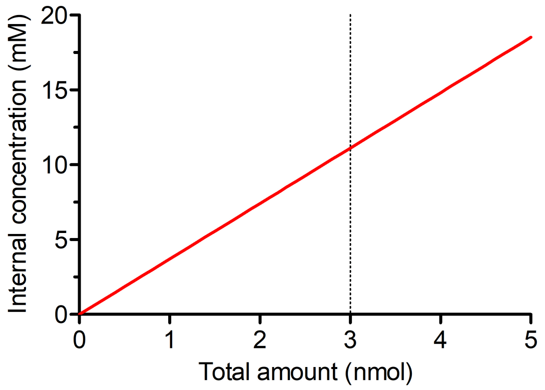

Figure S5 | Estimation of internal nucleotide concentration in the chemiluminescence assay. We assume an average specific internal volume of 2.7 µL/mg total lipids (18 µL per 6.66 mg total lipids)[2]. For ATP and total nucleotide quantification, samples of 100 µL of 2.7 mg/mL total lipids are diluted out to 160 µL, yielding 1.6875 mg/mL due to PCA and KOH/$KHCO_3$ addition. From this, a volume of 60 µL is used for ATP or total nucleotide determination, consisting of 0.1 mg total lipids and an in internal volume of 0.27 µL (0.45% vol/vol). The chemiluminescence assay yields 3 nmol of nucleotides, corresponding to an internal concentration 11.1 mM, which is in good agreement with the encapsulated concentration of 10 mM of ADP (given the uncertainty in the estimation of the specific internal volume of liposomes).

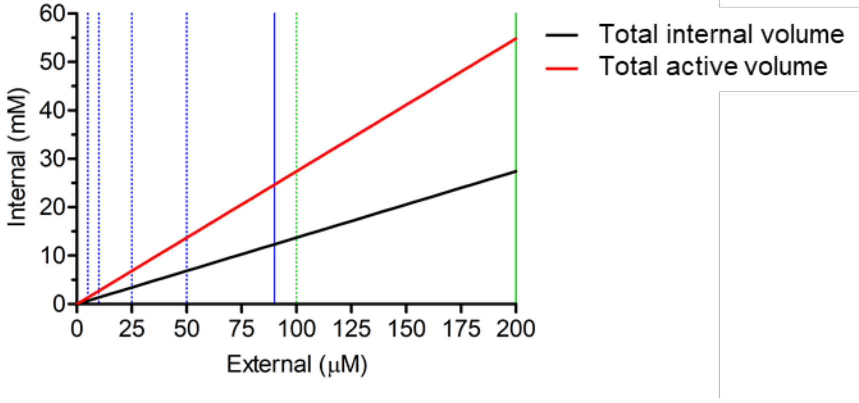

Figure S6 | Estimation of internal glycerol concentration. We calculate that samples of 120 uL and a total lipid concentration of 2.7 mg/mL (used for the online measurement of ATP/ADP) consist of 0.324 mg of lipids and an internal volume of 0.8748 uL (0.73% vol/vol). Thus, we apply a correction factor of 137 to determine the internal glycerol concentration (total internal volume, black slope). This correction factor may be further adjusted by taking into account that the "active" volume, *i.e.* the volume that contains all components and leads to a functional pathway, is smaller that the total internal volume due to all components not being present in all liposomes[6–8]. From the chemiluminescence experiments (that gave a 50% conversion, see Figure2e in the main text), we estimate an active volume of 0.5x the total internal volume, leading to a correction factor of 274 (active internal volume, red slope). In the glycerol titration experiment, 20-50 mM glycerol was cumulatively added to the samples (blue and green thick lines; dashed lines are single additions; see main figure), which is well in excess of the 10 mM ADP initially present. The correction factor of 274 also holds for the chemiluminescence experiments.

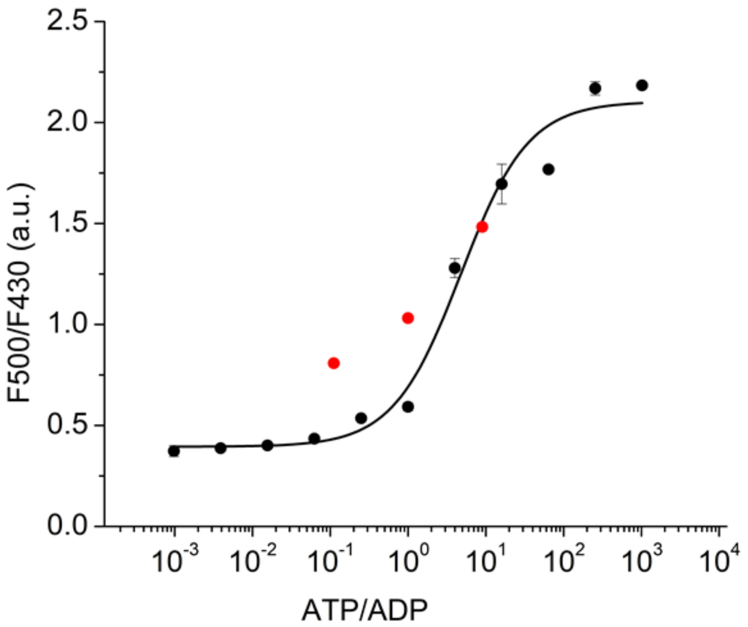

Figure S7 | Calibration curve of PercevalHR in solution and in liposomes. A calibration curve was prepared for PercevalHR in solution (black dots; n=2 and error bars represent s.d.) and in vesicles (red dots; n=2 and error bars represent s.d.). The in solution calibration curve was obtained by calculating the F500/F430 ratio from spectra acquired in the presence of different ATP/ADP ratios (with ATP plus ADP always equal to 10 mM). The curve was fitted with a Hill equation (n fixed to 1) and found to be in good agreement with previously reported curves[2]. The vesicle samples containing 1:9, 1:1 and 9:1 ATP/ADP (ATP plus ADP equal to 10 mM) were found to significantly deviate from the calibration curve in solution. Therefore, we report the F500/F430 values rather than ATP/ADP ratios.

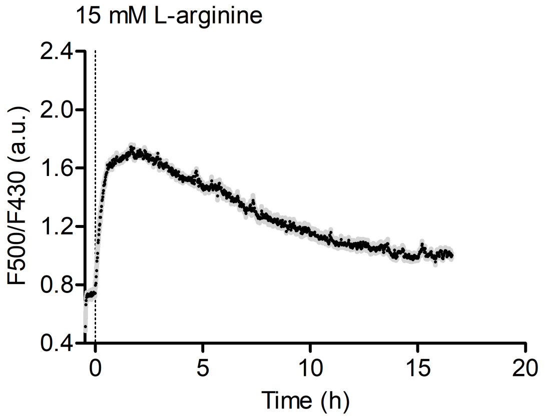

Figure S8 | ATP/ADP in the presence of 15 mM L-arginine. ATP/ADP levels were measured with PercevalHR in the presence of an excess of L-arginine. The vesicle composition was as reported in Figure 2a in the Main text, with the exception that arginine was 15 mM instead of 5 mM and no glycerol was added. Here, the ATP/ADP signal decrease is more prominent and it does not level off after 9 hours as observed with 5 mM L-arginine.

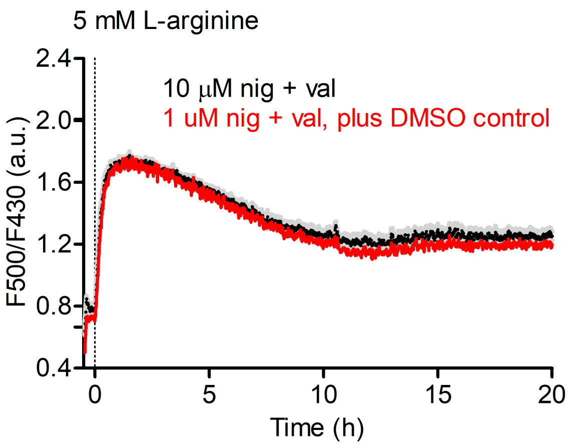

Figure S9 | ATP/ADP in the presence of a tenfold excess of valinomycin and nigericin (10 µM, 4% vol/vol DMSO). A control that the proton and potassium gradients are fully dissipated and do not affect the PercevalHR readout by altering the pH is taken by using a ten-fold excess of the ionophores nigericin and valinomycin. The vesicle composition was as reported in Figure 2a in the Main text, with the exception that nigericin and valinomycin were added to a final concentration of 10 µM (black trace; gives final 3.6% vol/vol DMSO) and that no glycerol was present. To control for undesired side effects due to the higher DMSO concentration, a control was taken with the standard ionophore concentration of 1 µM (red trace; gives final 0.4% vol/vol DMSO), to which 3.6% vol/vol DMSO was additionally added. We find that the ATP/ADP levels are comparable with 1 µM and 10 µM nigericin and valinomycin. In addition, the higher DMSO concentration did not have a significant effect on the vesicle stability.

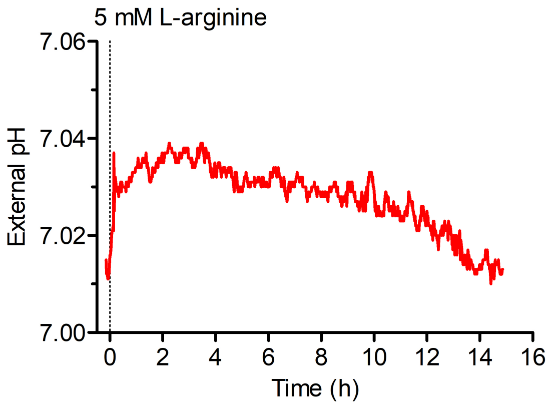

Figure S10 | pH of external medium over time. The external pH was measured with a pH electrode over time in 500 µL 2.7 mg/mL of vesicles to which 5 mM L-arginine was added. The vesicle composition was as reported in Figure 2b in the Main text, with the exception that no glycerol was used.
